# Acute exudative polymorphous paraneoplastic vitelliform maculopathy (AEPPVM) associated with choroidal melanoma

**DOI:** 10.1186/s40942-021-00300-0

**Published:** 2021-04-01

**Authors:** Aluisio Rosa Gameiro Filho, Guilherme Sturzeneker, Ever Ernesto Caso Rodriguez, André Maia, Melina Correia Morales, Rubens N. Belfort

**Affiliations:** Ophthalmology Department, Federal University of São Paulo (Unifesp–EPM), Rua Botucatu, 822, São Paulo, SP 04023-062 Brazil

**Keywords:** Maculopathy, Melanoma, Metastasis, Paraneoplastic, Vitelliform

## Abstract

**Background:**

To report a case of acute exudative polymorphous paraneoplastic vitelliform maculopathy in a patient with a history of choroidal melanoma, with metastases to the pancreas, liver, and central nervous system.

**Case presentation:**

A 63-year-old patient, with a history of enucleation of the right eye due to choroidal melanoma, complained of progressive visual loss during a follow-up visit. Fundoscopic examination revealed multiple small areas of serous retinal detachment scattered throughout the posterior pole and ancillary tests confirmed the diagnosis of acute exudative polymorphous paraneoplastic vitelliform maculopathy (AEPPVM). Screening for systemic metastases showed pancreatic, hepatic, and central nervous system involvement.

**Conclusions:**

We describe a rare case of acute exudative polymorphous paraneoplastic vitelliform maculopathy, which should be considered in patients with or without a history of melanoma, who have vitelliform retinal detachments. Nevertheless, no previous reviews of literature have shown a correlation between AEPPVM and pancreatic metastasis.

## Background

Paraneoplastic syndromes are defined as clinical conditions involving non-metastatic systemic effects that accompany malignant disease. They are believed to occur in 10% of cancer patients. Those involving the nervous and visual system are even less common, affecting 0.01% of patients in a tertiary Oncology center [1].

Paraneoplastic retinopathy is a heterogeneous group of disorders caused by the immunologic effects of extraocular cancer in the retina. Autoantibodies originated against distant tumoral antigens cross-react with retinal proteins, resulting in retinal damage and subsequently degeneration [2]. They include cancer-associated retinopathy (CAR), melanoma-associated retinopathy (MAR), acute exudative polymorphous paraneoplastic vitelliform maculopathy (AEPPVM), bilateral diffuse uveal melanocytic proliferation (BDUMP), and paraneoplastic optic neuropathy (PON).

CAR is the most frequent paraneoplastic retinopathy, usually seen in patients with small-cell lung carcinoma, but can also be associated with breast, gynecologic and other carcinomas. CAR is frequently related to autoantibodies against recoverin, α-enolase, and anti-transducin α [1, 3]. On the other hand, MAR is a rare condition, usually seen in patients with metastatic cutaneous melanoma, and, even more rarely, in patients with uveal melanoma. Originally described with a normal fundus appearance [2], more recent reports have described paraneoplastic conditions with several fundus abnormalities, such as AEPPVM. In this study, we report a case of AEPPVM in a patient with choroidal melanoma and systemic metastases.

## Case presentation

A 63-year-old male patient came for a follow-up visit at the Ocular Oncology Service of the Paulista School of Medicine (Federal University of São Paulo). He had been diagnosed 3 years before with choroidal melanoma in his right eye. At his initial evaluation, best-corrected visual acuity was hand motion in his right eye and 20/20 in his left eye. In the fundoscopy, he presented an inferior pigmented choroidal lesion in his right eye. His left eye was unremarkable and family history was negative. The ocular echography showed a dome-shaped lesion of 12.8 mm of thickness and a basal diameter of 15.9 mm, suggesting the diagnosis of choroidal melanoma. Enucleation of his right eye was performed. Pathology of the surgical specimen confirmed a choroidal melanoma, with the predominance of epithelioid cells, total retinal detachment, and superficial scleral invasion. Systemic evaluation showed no signs of metastasis. After that, the patient was followed-up with periodic abdominal image examinations (ultrasonography, tomography, or magnetic resonance imaging), serum liver function tests, and chest radiography, at a 6-month interval.

**Fig. 1:**
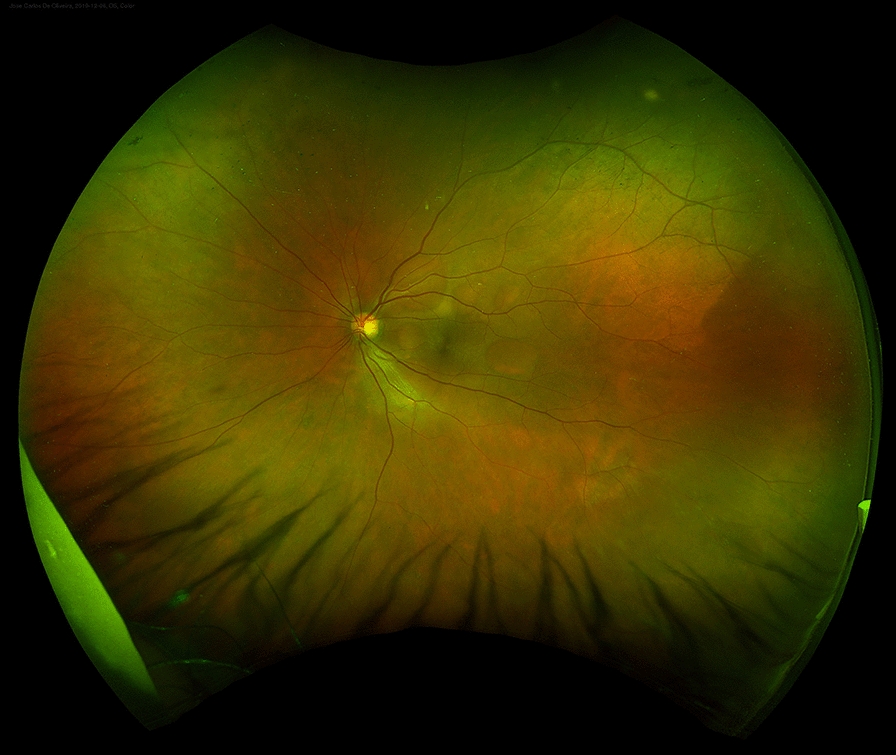
Fundus examination showing multiple small areas of serous retinal detachment throughout the posterior pole, with yellowish vitelliform lesions

**Fig. 2:**
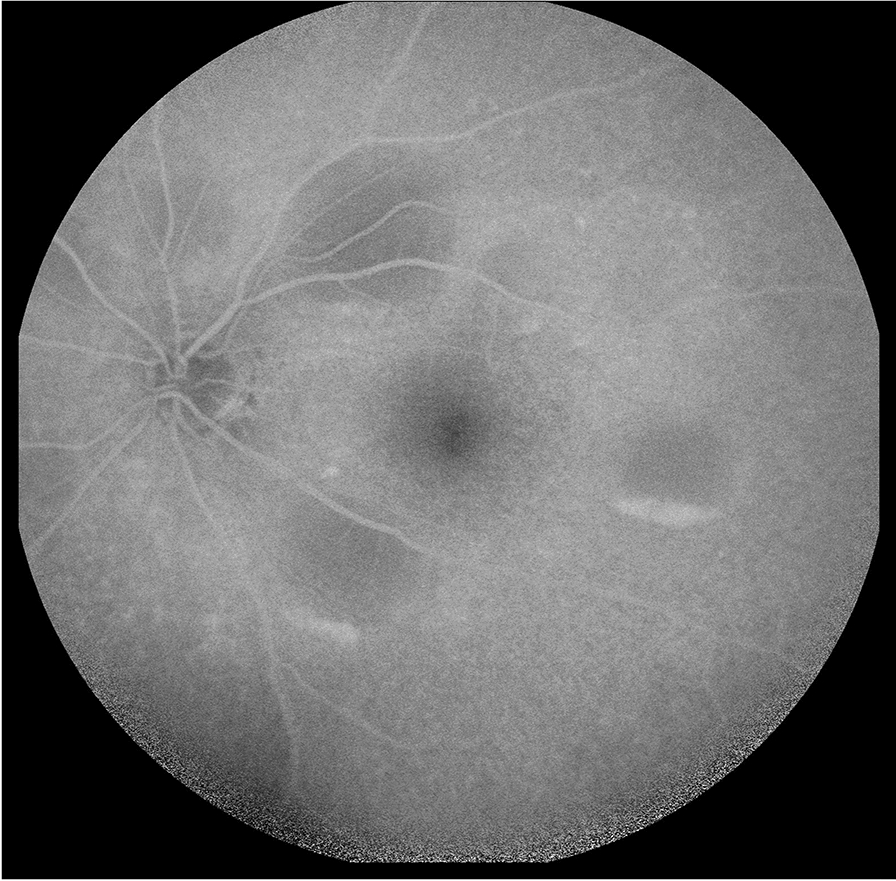
Fundus autofluorescence (FAF) showing hyperautofluorescent material at the inferior part of the lesions, with a pseudohypopyon configuration

**Fig. 3:**
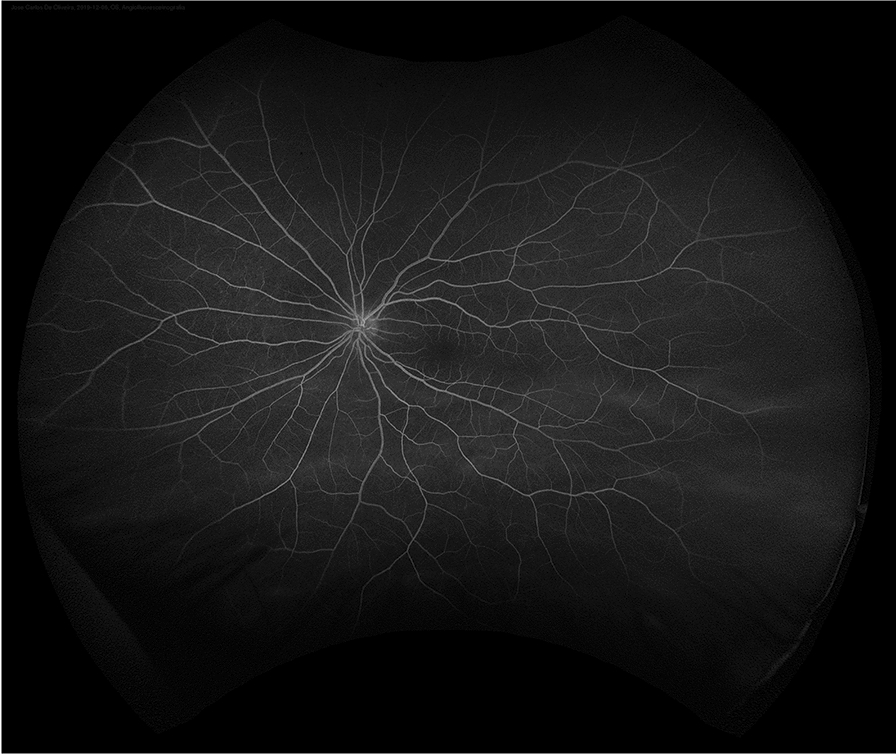
Fluorescein angiography (FA) showing a slight blockage of fluorescence and absence of late staining of lesions

**Fig. 4:**
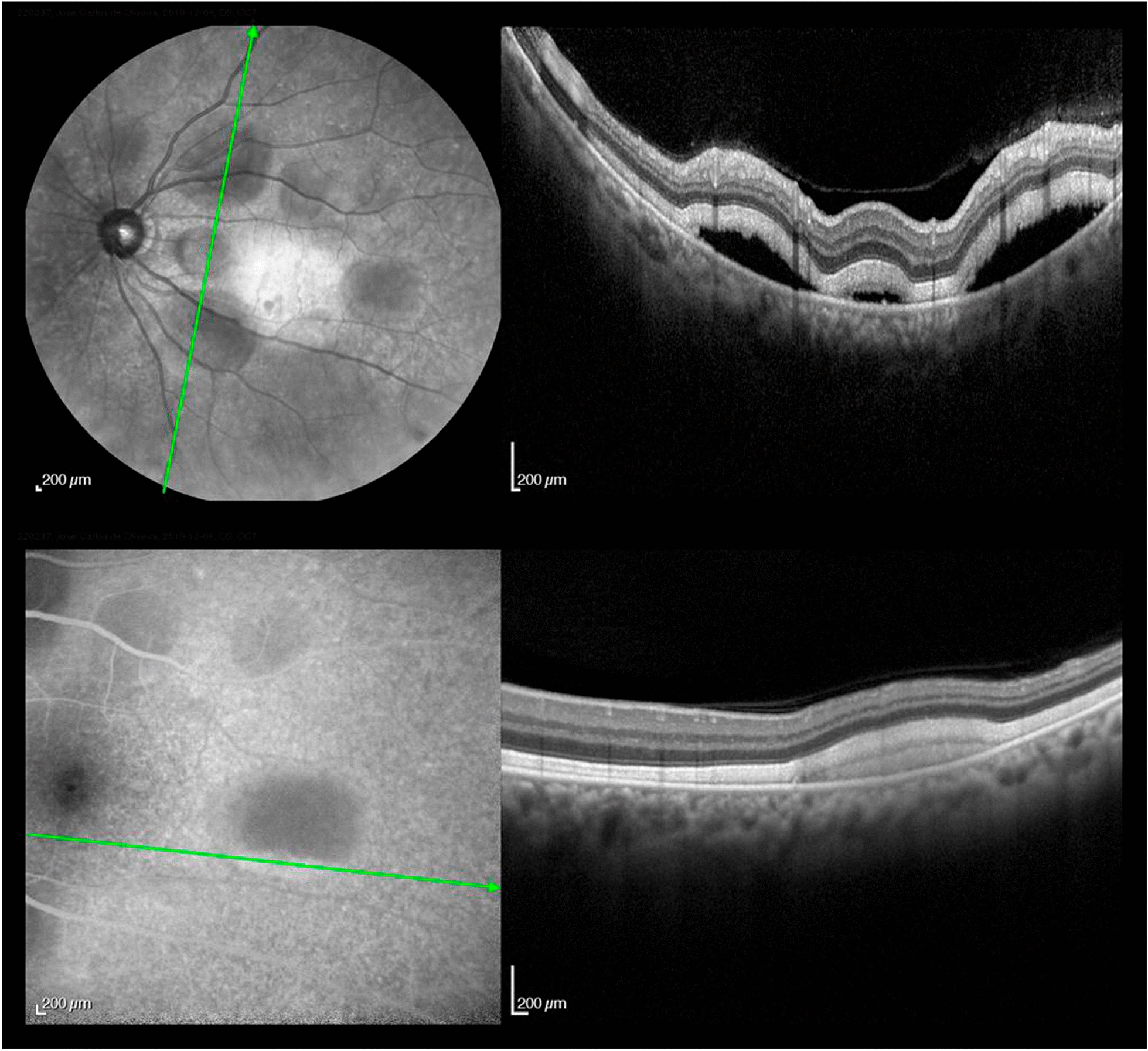
SD-OCT showing areas of subretinal fluid and normal choroidal thickness

At his routine check-up 3 years after the enucleation, he complained of progressive visual loss. His visual acuity was 20/30 in his left eye. Slit-lamp examination revealed mild nuclear sclerosis, intraocular pressure was normal and there were no cells in the anterior chamber or vitreous. On dilated fundus examination, multiple small areas of serous retinal detachment were noted scattered throughout the posterior pole, with yellowish vitelliform lesions (Fig. 1). Fundus autofluorescence (FAF) showed hyperautofluorescent material at the inferior part of the lesions, with a pseudohypopyon configuration (Fig. 2). On fluorescein angiography (FA), there was a slight blockage of fluorescence and there was an absence of late staining of lesions (Fig. 3). Spectral-domain optical coherence tomography (SD-OCT) showed areas of subretinal fluid, excluded choroidal involvement in all of the lesions, and showed normal choroidal thickness (Fig. 4).

**Fig. 5:**
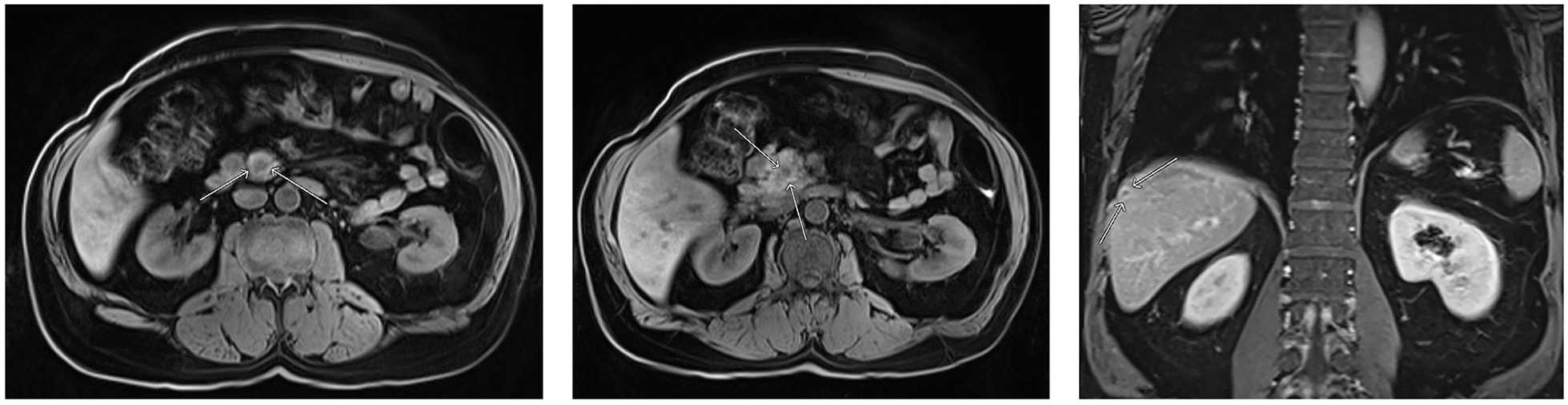
Abdominal magnetic resonance imaging showing: hypovascular nodule in the uncinate process (**a**) and in the pancreatic head (**b**), as well as a 0.9 cm lesion near the hepatic capsular region (**c**)

Abdominal magnetic resonance imaging showed two pancreatic nodules, one in the head, with 1.1 cm, and another in the uncinate process, measuring 1.5 cm (Fig. 5). Both nodules presented with low signal in T1 and restriction to diffusion, suggesting secondary neoplastic involvement. Endoscopic ultrasound-guided fine-needle aspiration of these pancreatic nodules was performed, showing epithelioid cells with melanin, nuclear hyperchromatism, evident nucleoli, and karyomegaly, confirming the hypothesis of metastatic melanoma. Image examinations also showed a 0.9 cm lesion near the hepatic capsular region with rim enhancement after contrast administration, believed to be another metastatic lesion (Fig. 5). The patient was referred to the Clinical Oncology Department for evaluation; however, he had a cerebrovascular accident, due to brain metastasis. His management is currently palliative.

## Discussion and conclusion

Dutch ophthalmologist Sotodeh [4] was the first to use the term paraneoplastic vitelliform retinopathy to describe three patients with a similar pattern of multiple scattered nummular yellowish-orange lesions at the retinal pigment epithelium (RPE) level in the posterior pole. Although usually associated with cutaneous melanoma, and less frequently with choroidal melanoma, it was previously reported in patients with non-melanoma malignancies, such as breast carcinoma, lung adenocarcinoma, and clear cell sarcoma [1]. It is currently under discussion if AEPPVM should be considered an atypical form of MAR or not.

AEPPVM is a rare condition: Grajewski[5] reviewed the data of 108 German patients with metastatic cutaneous melanoma and showed that only 2% of them had AEPPVM. According to current knowledge, fewer than 10 cases of AEPPVM associated with uveal melanoma have been previously reported [3–12].

AEPPVM is characterized by vitelliform yellowish lesions under the level of the neurosensory retina and RPE, associated with the accumulation of hyperautofluorescent material in the posterior pole and serous retinal detachment. It has an equal gender distribution, and the mean age at diagnosis is 59 years [1]. The onset of AEPPVM is usually remote from the primary tumor diagnosis: a review of previously published cases reveals that it ranged from 2 to 23 years after enucleation due to choroidal melanoma [6, 8]. Furthermore, AEPPVM frequently heralded metastatic spread [1], such as in the case presented in this manuscript.

Patients with AEPPVM can be asymptomatic or complain of nyctalopia, variable visual acuity reduction, visual field defects, shimmering photopsias, glare, and halos. Funduscopy reveals variable degrees of multifocal yellowish vitelliform lesions under the level of neurosensory retina, often associated with overlying serous detachment, with a tendency of the vitelliform material to gravitate in inferiorly and layer out within the areas of subretinal fluid [1]. FA reveals blockage of choroidal fluorescence and FAF demonstrates the corresponding hyperautofluorescence of the lesions. OCT demonstrates areas of localized serous retinal detachments and the absence of choroidal thickening, helping to exclude choroidal metastasis. The advent of SD-OCT also helped to accurately locate the accumulation of the vitelliform material and the fluid to the subretinal space, and not beneath RPE, as it was initially suggested [1]. Eletroretinogram abnormalities, such as a-wave and/or b-wave reductions can also be present, howbeit, with variable results [13].

Although the pathogenesis is not completely understood, some auto-antibodies have been related to this condition, such as anti-interphotoreceptor retinoid-binding protein (IRBP), peroxiredoxin 3 (PRDX3), bestrophin 1, carbonic anhydrase II (CAII), bipolar cells, and anti-transient receptor potential M1 (also known as melastin 1) [12].

AEPPVM is generally a bilateral condition, except for enucleated patients. Nagiel reported unilateral involvement in a patient who had been submitted to iodine-125 brachytherapy due to choroidal melanoma. In that eye, the absence of AEPPVM could be explained by extensive photoreceptor loss secondary to radiation, which precluded the aggregation of vitelliform debris [12].

Currently, there is no treatment for this condition. Immune system modulation with the use of steroids or immunosuppressive drugs is usually ineffective. Clinicians should be aware of and perform investigations for systemic involvement in patients with AEPPVM. This condition is usually indicative of poor prognosis, with most patients reportedly dying due to metastatic disease within a few months up to 4 years after initial presentation [1].

Differential diagnoses include acute exudative polymorphous vitelliform maculopathy (AEPVM), a clinically indistinguishable condition, characterized by acute bilateral visual reduction, and multiple round hyperautofluorescent yellowish subretinal lesions, usually preceded by headaches, upper respiratory syndromes, or flu-like symptoms. However, this condition is not associated with malignancies and has been reported in association with trauma and infeccious diseases, such as hepatitis C, Coxsackie B virus, syphilis and Lyme disease. Besides, AEPVM tends to recover spontaneously within several months [13]. Bilateral diffuse uveal melanocytic proliferation (BDUMP) is defined by early multiple round orange patches at the RPE level, and, in later stages, elevated uveal melanocytic lesions. This condition has never been reported in association with uveal melanoma. Another condition that could resemble AEPPVM is adult vitelliform macular dystrophy (AVMD), in which the patient has generally a single vitelliform lesion in the fovea.

Our patient has developed pancreatic metastasis from choroidal melanoma, which is also considered very rare. Usually, only 2% of pancreatic cancers are metastases from other primary sites [14]. Until the present date, there have only been six previous reports of pancreatic metastasis due to choroidal melanoma [15]. This is the first time AEPPVM is reported in association with pancreatic metastasis.

Management of paraneoplastic retinopathy remains difficult because of its rarity and unclear diagnostic criteria. Clinical suspicion remains the most important factor in the diagnosis. The presence of vitelliform lesions should alert the physician to the possibility of AEPPVM. Systemic investigation for metastatic spread must be undertaken, regardless of how long the patient has been in remission.

## Data Availability

All data generated or analyzed during this study are included in this manuscript.

## Abbreviations

AEPPVM: Acute exudative polymorphous paraneoplastic vitelliform maculopathy
CAR: Cancer-associated retinopathy
MAR: Melanoma-associated retinopathy
BDUMP: Bilateral diffuse uveal melanocytic proliferation
PON: Paraneoplastic optic neuropathy
RPE: Retinal pigment epithelium
FAF: Fundus autofluorescence
FA: Fluorescein angiography
SD-OCT: Spectral-domain optical coherence tomography
IRBP: Anti-interphotoreceptor retinoid-binding protein
PRDX3: Anti-peroxiredoxin 3
CAII: Anti-carbonic anhydrase II
AEPVM: Acute exudative polymorphous vitelliform maculopathy
